# Coordination of care for multimorbid patients from the perspective of general practitioners – a qualitative study

**DOI:** 10.1186/s12875-019-1048-y

**Published:** 2019-11-20

**Authors:** Judith Stumm, Cornelia Thierbach, Lisa Peter, Susanne Schnitzer, Lorena Dini, Christoph Heintze, Susanne Döpfmer

**Affiliations:** 1 Institute of General Practice, Charité – Universitaetsmedizin Berlin, Charitéplatz 1, 10117 Berlin, Germany; 2 Institute of Medical Sociology and Rehabilitation Science, Charité - Universitaetsmedizin Berlin, Charitéplatz 1, 10117 Berlin, Germany

**Keywords:** Primary health care, Multimorbidity - coordination of care - general practitioner, Medical practice assistant - navigator - qualitative research

## Abstract

**Background:**

In Germany, a decreasing number of general practitioners (GPs) face a growing number of patients with multimorbidity. Whilst care for patients with multimorbidity involves various healthcare providers, the coordination of this care is one of the many responsibilities of GPs. The aims of this study are to identify the barriers to the successful coordination of multimorbid patient care and these patients’ complex needs, and to explore the support needed by GPs in the care of multimorbid patients. Interviewees were asked for their opinion on concepts which involve the support by additional employees within the practice or, alternatively, external health care professionals, providing patient navigation.

**Methods:**

Thirty-two semi-structured, qualitative interviews were conducted with 16 GPs and 16 medical practice assistants (MPAs) from 16 different practices in Berlin. A MPA is a qualified non-physician practice employee. He or she undergoes a three years vocational training which qualifies him or her to provide administrative and clinical support.

The interviews were digitally recorded, transcribed and analysed using the framework analysis methodology.

**Results:**

The results of this paper predominantly focus on GPs’ perspectives of coordination within and external to general practice. Coordination in the context of care for multimorbid patients consists of a wide range of different tasks. Organisational and administrative obstacles under the regulatory framework of the German healthcare system, and insufficient communication with other healthcare providers constitute barriers described by the interviewed GPs and MPAs. In order to ensure optimal care for patients with multimorbidity, GPs may have to delegate responsibilities associated with coordinating tasks. GPs consider the deployment of an additional specifically qualified employee inside the general practice to take on coordinative and social and legal duties to be a viable option.

**Conclusions:**

The cross-sectoral cooperation between all involved key players working within the healthcare system, as well as the coordination of the whole care process, is seemingly challenging for GPs within the complex care system of multimorbid patients. GPs are generally open to the assignment of a person to support them in coordination tasks, preferably situated within the practice team.

## Background

Due to a recent demographic change in Germany, GPs are faced with a growing number of patients with multimorbidity. The care of these patients with complex needs is often provided by multiple doctors and a range of other healthcare professionals, and the coordination of their care is challenging for both GPs and patients [1]. Coordination of care for multimorbid patients means a functioning trans-sectoral care that interlocks all involved healthcare providers. The coordination of care especially includes work beyond the direct patient contact. The elements that define coordination are difficult to specify, because coordination tasks are not detached from other care tasks [2]. Research findings suggest that coordination of care in general practice as well as care provided by multidisciplinary teams can enhance the continuity of care of patient with multimorbidity [2–4].

Primary health care (−teams) play a decisive role in the coordination and therefore the continuity of care of multimorbid patients [2]. GPs provide comprehensive long-term care for patients with multimorbidity, playing the central role in patients’ care, which implies a sense of affiliation [5]. Often, they have to assume a mediating role between specialists and patients in order to find a compromise between the specialist’s recommendations and the patient’s needs, views and wishes [2–6].

At the same time, the decline in the number of GPs in Germany means that new concepts have to be developed to support them, particularly in the coordination of care for patients with complex needs [7].

Possible concepts include the integration of general practice into a cross-sectoral model of care [8], or the deployment of additional healthcare professionals to coordinate the care of patients with multimorbidity in general practice. The deployment of non-medical professions for coordination tasks and specific clinical duties could relieve the GPs and raise consultation times [2, 8, 9]. Furthermore, medical settings, in which physicians and non-medical healthcare professions work together in teams demonstrate improved patient outcomes [10]. A shared definition of goals, cooperative tasks and responsibilities help to enhance patient care [11].

In Germany, general practices are mostly small units with one or two GPs and two to four MPAs [9]. Currently, single-handed practices are the most strongly represented in Germany [12]. In general practices, the “qualified medical practice assistant” (“Medizinische Fachangestellte”, MPA), the primary mid-level healthcare professional, is an important member of German GPs’ practice teams. MPAs complete a three-year training programme which qualifies them for office management/clerical administration (such as scheduling appointments and updating patients’ files) and a broad range of nursing-based clinical duties (such as taking medical histories, explaining treatment procedures, performing diagnostic procedures like ECG and blood pressure measurements, taking blood samples, and providing home visits). Two thirds of the training programme take place in the practice and one third in vocational school [13]. Even though GPs have to provide medical treatments personally, they are allowed to delegate certain tasks. These tasks are defined in the “Agreement on the delegation of medical services to non-medical staff in ambulatory healthcare” by the Federal Medical Association [14]. Studies have shown that care management interventions are feasible and effective if they are delivered by MPAs [15, 16]. The involvement of MPAs can also improve patient care, and lead to a reduction of GPs’ workloads [17]. GPs and MPAs work in close collaboration, and MPAs enjoy a high level of acceptance on the side of patients [18].

In other countries, the involvement of additional health professionals in primary care, such as physician assistants, nurses and nurse practitioners in anglo-american countries or “praktijkondersteuner” in the Netherlands is common. A multidisciplinary primary care team might contribute to more comprehensive patient care and improve the coordination of care in comparison to a physician working in a solo practice (Bellagio-Model) [19].

Another model for the improvement in the care of patients with complex needs is based on navigation. Patient navigation is an umbrella term based upon models of care and case management that include support and guidance for patients accessing the care system [20]. Patient navigation does not address a single point in care but follows patients proactively throughout the entire process of ambulatory and inpatient healthcare. Navigation programs differ in types of navigator roles; common tasks are the provision of information, guidance, advocacy, emotional and practical support, as well as education and empowerment [20, 21]. A central aim of patient navigation is to serve vulnerable populations [20].

The research consortium “NAVICARE – Patient-centred health services research” (https://navicare.berlin/de/), funded by the Federal Ministry of Education and Research (“Bundesministerium für Bildung und Forschung”, BMBF), aims to acquire knowledge about the barriers which keep patients from receiving optimal care, and to study effective, patient-oriented ways to overcome these barriers, such as navigation, by exploring different perspectives in two subprojects, see Additional file 1: Figure S1.

The subproject COMPASS (https://navicare.berlin/de/forschung/navicare-forschung/) focuses on the perspective of the GP practice and patients in the long term ambulatory care. This article concentrates on the findings of the first stage of the mixed-methods approach within COMPASS, the interviews with GPs and MPAs. The aims of the interviews were 1.) to explore the views of GPs’ and MPAs’ on requirements and barriers for the successful coordination of care for patients with multimorbidity and complex needs, 2.) to identify the need for support, and 3.) to investigate opinions on two different models of support: support from within the practice, by delegation and/or expansion of the practice team, or support from external health care professionals, providing navigation.

## Methods

We conducted semi-structured interviews with 16 GPs who are statutory health insurance registered (“KV-Ärzte”) in Berlin, as well as one of their MPAs (*n* = 16). The participants were purposively sampled from the practice-based research network “ANCHOR” of the Institute of General Practice, Charité - Universitaetsmedizin Berlin. They were approached via e-mail and telephone. The selection of GPs was carried out in order to represent the Berlin GP population in terms of gender as well as the maximum spread of practice locations over different city districts of Berlin. The GPs then designated one MPA from their practice as the other interview partner, meaning that from each recruited practice, one GP and one MPA were interviewed individually. The interviews took place in the respective GP practices.

Interviews were audio-recorded and transcribed verbatim. Any identifying details were removed and participants were assigned pseudonyms. Written informed consent was obtained from all participants.

The study was approved by the research ethics committee of the Charité - Universitaetsmedizin Berlin (EA4/034/17).”

The interview guideline was developed based on studies from the previous literature as well as the experience of the researcher. It was discussed in a multidisciplinary working group and adjusted until a consensus was reached. The same interview guideline was applied to GPs and MPAs. The interview guideline included a number of open questions regarding the theme’s coordination, delegation, optimal care and community care in general practice (see Table 1).

**Table 1:** Topic guide with exemplary questions from the interview guideline

Theme: Coordination	
Topic	Examples of questions
1. Problems/Barriers in health care coordination	Can you recall a multimorbid patient of yours with complex care needs?
2. Patient groups with need for coordination	Are there any other patient groups that you think are particularly affected by coordination difficulties?
3. Achievements in the coordination of health care	Can you think of an example where coordination went well?
4. Improvements in the coordination of health care	You mentioned some problems and achievements, do you have additional suggestions on what you would like to improve in coordination?
5. Coordination within your practice	Could you imagine that one or more people in your practice take on coordinative tasks?
6. Coordination offers outside the general practice	There are concepts and projects that introduce so-called extern navigators - these are additional people who are supposed to take on specific coordinative tasks but are not permanently located in the GP practice. Do you have any experience with this?
Theme: Delegation	
7. Delegation of medical services from doctor to medical practice assistant in the practice	Which additional tasks would you hand over to your medical practice assistant?
8. Recognition of the professional group of the medical practice assistant	What could be an appropriate appreciation/compensation for the additional services and responsibilities of the medical practice assistant?
9. Team collaboration	How do you exchange information about patients in your team?
10. Multidisciplinary collaboration	Could you imagine having an employee in your practice who is neither a medical doctor nor a medical assistant, but belongs to a new professional group and takes on tasks of care and coordination?
Theme: Optimal Care	
11. Definition of optimal patient care from a primary care perspective and viewpoint of the MPA	What would optimal care mean for your sample patient from the question in the beginning?
Theme: Community Care	
12. Use of social and medical support services in the region	What are your experiences regarding medical and social support services? Which ones do your patients use?

Topic guide with exemplary questions. Complete interview guideline is available from the authors upon request. Questions could be individually adapted to the conversation flow of the respective interviews

Face-to-face interviews were chosen as the data collection method for the first stage of the COMPASS project. This was in order to ensure in-depth discussions of this complex topic. The issues raised in the interviews were then used to develop a questionnaire that was sent to all GPs in Berlin who are statutory healthcare registered. The aim was to supplement the qualitative study results by obtaining a broader perspective of a large number of GPs. In this paper we report the results of the interviews with the GPs, complemented by the views of the interviewed MPAs.

### Data analysis

By using the framework analysis [22] as a systematic approach to manage and analyse qualitative data, seven steps have been implemented: (1) transcription of the interviews; (2) familiarisation with the interviews; (3) coding; (4) developing a working analytical framework; (5) applying the analytical framework; (6) charting data into the framework matrix; and (7) interpreting the data. The interviews were analysed by a research team that included researchers from different professional disciplines, such as GPs, health services researchers, a sociologist and a public health scientist. By applying these different perspectives, researcher (investigator-) triangulation was achieved. Two researchers (CT, JS) coded the interviews independently. Subsequently, both researchers compared, discussed and, if necessary, adjusted the coding. A combined deductive and inductive approach was used for the coding procedure. Consensus on the interpretation was established in the group of researchers after a thorough discussion of the data.

All interviews were analysed with the support of the qualitative analysis software program MAXQDA® 18 (VERBI software GmbH, Germany).

## Results

We interviewed 16 GPs from nine different city districts and, independently, 16 MPAs from the same practices (for details, see Table 2). Each interview lasted 45 to 60 min.

**Table 2:** Characteristics of interviewees by group (*n* = 32)

Characteristics of interviewees		
	GPs (n = 16)	MPAs (n = 16)
Gender	Male: 10	Male: 2
	Female: 6	Female: 14
Age (years)	Median: 54.5 (Range: 39–69)	Median: 44 (Range: 21–63)
Practice type	Single handed: 7	
	Group: 9	
Number of employees in the practice	Median: 5.5 (Range: 3–19)	

### Coordination from the perspective of the GPs

Initially, we wanted to know what the term “coordination” means to GPs in the context of care for patients with multimorbidity. The interviewed GPs had various opinions on what coordination consists of.

*“Well everything I do here is actually coordination, I bring the various support possibilities and healthcare services together, yes. I do little else except coordinate. Apart from prescribing tablets. [ …*] *and so we actually do very little. We are more a sort of dispatcher; we connect where and when it makes sense.” (GP_10).*

*“[ …*] *Coordination means: one learns from the other … and one informs the other, like hospitals and GPs. [ …*]” *(GP_11).*

*“Determining the medication regime; ensuring home care; referrals, also for physiotherapy, logotherapy etc.; avoiding hospitalisations; and the quality of life of the patient and the psychosocial context are also important.” (GP_15).*

The coordination role of the GP starts with being the primary contact for the patient, assessing the needs of the patient and their relatives and organising further help structures.

The general practice constitutes a social and medical contact point and simultaneously provides immediate and sustained medical care. It can act as an advisory point for medical matters but also for social and legal problems, relating to questions relevant to family as well as local community relationships, and questions relevant to social legislation like gaining access to social benefits and socio-medical services.

*“[ …*] *The general practitioner is the person who is always in charge.” (GP_7).*

GPs perceive themselves as the appropriate person to coordinate patients’ care. They take on a coordinating role by law (Book V of the Social Law § 73), but also see themselves as the authority to manage the patient’s entire care process. The role of the coordinator includes organisational and administrative matters, as well as coordinating care involving other medical and non-medical professionals, such as other medical specialists, home care services, physiotherapists, psychologists or health insurance.

GPs cite examples of successful coordination, such as the involvement of patients’ relatives, the implementation of the standardised national medication plan, the introduction of disease management programs (DMP), as well as quality assurance measures.

In summary, from the GPs’ perspective, good coordination:

- requires defining what is best for the patients and acting in the interests of the patients;
- in general practice, is based on reliability as well as trust in the practice staff;
- requires that all medical and non-medical key players engaged in the patient’s care have knowledge of and communicate with each other.

### Barriers to successful coordination

Frequently reported problems in the coordination of patients’ care were constraints resulting from administrative and organisational issues, the regulatory framework for GPs in Germany, and the communication with other health professionals and specialists.

#### Organisation and administration

Many of the patients’ needs are not of a medical nature. Some of the interviewed GPs feel that there is a need to delegate services that do not fall within their responsibilities to other professionals. These needs are not of a medical nature and rather relate to social and legal issues.

*“[ …*] *There is a need for services that essentially don’t fall into the responsibility of the GPs because they are not of a medical nature. They could be delegated to a person who is qualified in social matters, for example, social services.” (GP_16).*

Many interviewed GPs feel burdened by the lack of time in the care of patients with complex needs. The scheduled time for consultation does not provide enough time to deal with the multiple issues of the patient.

*“Lack of time is the biggest problem. The patients have too many issues … and after the consultation, they don’t know any more what has been discussed. I try to write it down for them, but I only have the time for very short notes. [ …*]” *(GP_8).*

The burden of administrative and organisational requirements seems to be particularly high when caring for patients with multimorbidity.

According to the interviewed GPs, organisational and administrative tasks include accounting, arranging appointments with specialists and other healthcare providers, filling out and signing forms (such as assessment forms for nursing care), organising home care services, and organising the transfer of immobilised patients to hospitals or other health specialists.

*“Let me put it this way. We have an 85-year-old male patient; we have to arrange appointments for him, organise transport and even have to print out his medical report and make sure he takes it with him.” (GP_5).*

Some GPs feel that certain organisational duties could be assigned to a trusted MPA, but most GPs want to retain ultimate responsibility. Others would appreciate an additional employee to take responsibility for all future organisational and administrative tasks.

*“I would wish that all these forms […*] *could be signed by a nurse,* e.g. *for home care, ergotherapy, even the repeat prescriptions. Why not? This would be a relief for me because for this you don’t need to be a doctor […*] *But this isn’t possible with the existing bureaucratic regulations.* “*(GP_1).*

#### Regulatory framework for GPs in Germany

Individual elements of the general framework of the German health system seem to cause a barrier to patient care.

The interviewed GPs reported that they need to justify necessary care beyond the basic level of services, which causes extra administrative work.

In this context, a frequently mentioned problem is the insufficient renumeration for the care of patients with complex and frequent needs. GPs describe that they are often in conflict in terms of whether to act in line with economic considerations or to offer optimal care for this patient group by investing a considerable amount of time and financial resources.

*“[ …*] *The lump sum payment per patient per three months only covers two visits per patient. Patients who are chronically ill and have to come more often are not sufficiently covered. [ …*] *If I wanted to work economically, I could only see the patients twice every three months. But even if the patient is not here, I still have to react to diagnostic reports and have to organise things like transports to other doctors or arranging appointments [ …*] *If I want to care for the patients at the highest possible level, I have to do that for free.” (GP_5).*

In Germany, every patient has to be personally seen by the doctor, even for minor ailments. Some interviewees believe that an employee with an additional qualification in certain fields could relieve the GP and provide more room for the care of patients with complex care needs.

*“This would indeed be a relief for us, but it would also turn our system on its head (laughs). We are a doctor-dominated system and every patient with a bug, such as a cold, has to visit a GP. Of course, it would make more sense if not every patient with minor ailments was obliged to visit a GP; why should I as a doctor see every patient with a cold? It’s completely absurd. Economically, of course, it makes sense: financially it is attractive to see healthy patients.” (GP_12).*

In Germany, GPs don’t have a gatekeeping function. Therefore, some patients don’t even have a GP. Most of the interviewed GPs perceived that multimorbid patients with insufficient social support from a stable social environment who are not in contact with a general practice, are in danger of slipping through the net of the German healthcare system. On the other hand, patients with complex needs may make extensive use of the system. For both patient groups, it would be helpful if someone led them through the “jungle” of manifold available healthcare resources and coordinated their care.

*“This is a structural problem: someone rambles through the health care system without any coordination, then suddenly hits the GP practice with a completely unclear situation. Then it is impossible to reconstruct the story because reports were not collected anywhere” (GP_12).*

#### Communication with external healthcare providers

The interviewed GPs reported a lack of sufficient communication structures with other caregivers such as specialist physicians, home care nurses or physiotherapists. Much time has to be invested into trying to contact other healthcare providers in order to coordinate the next steps in the care of the patient. In Germany, patient data is not exchanged electronically between healthcare professionals. External patient documents such as discharge letters and findings are usually sent by post.

Often medical reports are only sent to GPs after repeated reminders. As a result, the coordination of care becomes difficult when important information is missing or is difficult and time-consuming to obtain.

*“I actually don’t spend most of my time treating patients and writing prescriptions; I spend most of my time talking, listening, calling someone, not being able to reach someone and then having to call someone else. This makes some situations very difficult.” (GP_14).*

The GPs reasoned that better cooperative treatment with the possibility of easily accessible, specialised counselling for patient-related issues would lead to better patient care.

In the interviews, they often emphasised the difficulty that patients have obtaining appointments with specialists, as well as the lack of opportunities for professional exchange with other doctors.

*“To obtain an appointment with a specialist sometimes is really difficult for the patient. Principally, it is an option that we as GPs try to arrange for an appointment, but we also have to phone several times before we reach somebody, and this is extremely time-consuming and often unsuccessful. […*] *So, you have to send the patient home and try it again after office hours, and finally you have to ask the patient to come again to your practice to explain everything. All this requires considerable effort …* “*(GP_3).*

#### Discharge from hospital

Another frequently mentioned problem is the uncoordinated management of discharge from hospital. Often the patient gets discharged from hospital without ensuring the further continuation of treatment. The GPs don’t receive information about the patient’s discharge in advance to prepare further care. Often discharge takes place just before the weekend and without sufficient medication.

*“I believe that patients are getting pushed out of clinics. From a GP’s perspective, we are being presented with a fait accompli without being consulted. And even as GPs, we can’t organise home care on a Friday evening. The cooperation just doesn’t work.” (GP_2).*

Through premature and/or uncoordinated discharge from the hospital, aftercare is shifted from the hospital to the general practice.

### Concepts for task shifting to an additional healthcare professional inside or outside the general practice

During the interviews, the GPs identified some tasks and responsibilities that, in principle, could be shifted to other professional groups inside or outside general practice (see Table 3). From the perspective of the GPs, it is an essential prerequisite for task shifting that the patients are open to a new person becoming involved in their care. The patients need a person whom they can trust.

**Table 3:** Possible areas of responsibilities for an additional health care professional

Arrangement of appointments with specialists	
Organisation of the patient transfer	
Providing advice relating to social and legal services	
Undertaking home visits	
Coordination of patients with dementia or multimorbidity	
Contact and communication with other health care professionals involved in the care	

During the course of the interview, the GPs were asked for their views on two different concepts to support the general practice in the coordination of care: the deployment of an external navigator who is not affiliated with the general practice; or, alternatively, the employment of a further healthcare professional within the practice.

#### Deployment of an external navigator

The interviewer explained the definition of an external navigator as a person who is not employed by the practice but is affiliated to another institution that participates in the collaborative care of the patient.

Only very few of the interviewed GPs had ever heard or read about the possible role of a “navigator”, and most of them were sceptical. Initially, they could not imagine which professional group had to the skills and abilities to work as an external navigator. For GPs, a well-coordinated team is crucial. An external person, who does not know the team members and the work structure of the practice, could be a disruptive factor. The GPs feared needing to invest even more time and energy into the integration of yet another person who does not know the work processes of the practice.

*“( …*) *No, I haven’t had any experience with it at all. I think it’s a waste of time and energy. I can’t see how this cooperation can work out; it would be like Chinese Whispers. There will be yet another person between me and the patient. I’d prefer to deal directly with the patient or ask my MPA to sort things out. I think it will just cause further mistakes and I don’t have the time. When it comes to time management, I can’t see how it would improve anything either.” (GP_14).*

Table 4 gives an overview of the reasons GPs are sceptical about both task shifting concepts or why they refuse to work with an external navigator or an additional employee inside the general practice.

**Table 4:** Arguments against a task shifting concept

Limited financial resources	
Requires a large amount of time	
MPAs take over most of the tasks already	
MPAs are familiar with the patients	
Deployment location only possible outside the practice, for example, in home care	
Patients need a person they know	

A few GPs still felt attracted by the idea of an external navigator, although they remained sceptical at the same time.

*“Yes, for the moment, as an interim solution, this might be a good idea. But I think in the long run, another institution can’t replace what should actually happen in the practices. [ …*] *And there are too many multimorbid patients. So, this would only work in lighthouse projects but could not be applied comprehensively. [ …*]” *(GP_12).*

#### Deployment of other healthcare professionals inside the practice

The idea of the employment of a further healthcare professional within the practice was accepted more openly than the idea of an external navigator. From the perspective of some of the interviewed GPs, shifting tasks and responsibilities to an MPA could be a possible solution, either with an MPA who already is a member of the team or to an additional MPA.

*“The point is that the medical practice assistants know the patients, and this, of course, gives them an enormous advantage over external professionals.” (GP_13).*

The idea of assigning a person who works within the practice and who is familiar with social and legal matters was mentioned frequently by the GPs.

*“Anyone medical, social, possibly social AND medical, so that they can take part in the process. In an ideal world it would be a medical practice assistant or a nurse or even better, a medical practice assistant who has had further training.” (GP_11).*

Most of the interviewees felt that an additional person involved in the care of multimorbid patients inside the practice could reduce the burden of the GPs as well as MPAs, given that the effort required to involve this person is low.

#### Social worker/ person who is familiar with social and legal matters

GPs are highly aware of the patients’ unmet needs with regards to social and legal issues, especially in the context of multimorbidity. Upon asking the GPs which professional group would be the most appropriate to meet these needs, the majority of GPs mentioned the need for a qualified person to take care of all social and legal demands.

*“Well I would like it if I had someone who could take over the social issues, a kind of social worker. There are lots of problems which older patients have and can’t deal with any more, such as writing applications and filling out forms. I would like it if there was someone here who could explain how to draw up a living will. I just don’t have the time and neither do my MPAs, so I can’t delegate it to them.” (GP_9).*

*“I don’t know how other doctors manage, but I think they face the same difficulties. I have problems with all this social legislation, I never really got to grips with. This gets more and more complicated. [ …*] *How is a physician sitting in his practice all day long supposed to understand this? If this could be done by someone else …*” *(GP_1).*

Several possible ideas for assigning social and legal matters to an additional member of the care team were discussed. These consisted of:

- A social worker who is employed within the general practice, belonging to the established practice team.
- A social worker who is not directly affiliated with the general practice, but to another institution.
- A social worker who is shared by several general practices in the district and who could be deployed to different practices on an hourly basis.
- Qualifying the existing MPA with training or specialisation in social and legal affairs. However, to ensure financing, additional services must be covered by the public health insurance scheme.

The role of the social worker is primarily to be the first contact person for patients, relatives, GPs and other relevant parties with regards to social and legal issues.

### Perspective of the MPAs

MPAs perceived similar problems to GPs in the coordination of care of multimorbid patients. Organisational and administrative issues appeared to be a major concern and require additional time for MPAs as well as for GPs.

The MPAs described themselves as the principally responsible person for administrative and organisational tasks. Phone calls, arranging appointments and filling in forms are typical administrative and organisational tasks for MPAs.

*“It is really time-consuming to arrange appointments and organise the transport of immobile patients. [ …*]” *(MPA_7).*

By assigning the full responsibility for these matters to MPAs, in their view, double structures in administrative procedures could be avoided.

*„ […*] *As soon as the request for nursing care is received it is processed by us. Then the doctor checks it back; he determines whether everything is filled in correctly. Finally, he signs it and gives it back to us to send the formula back to the patient or nursing care.” (MPA_15).*

In contrast to the GPs, the MPAs tended to focus more on the coordination within the general practice and the cooperation of everyone involved. For example, the issues of priority were the communication with the patient and their relatives as well as the regular scheduling of patients’ appointments.

*“Important is the cooperation between my boss and us, and with the relatives; only if we all have the same goal can things work out”. (MPA_9).*

The interviewed MPAs were highly motivated and willing to extend their knowledge by undergoing further training. Nevertheless, a lack of time was considered to be a barrier to acquiring additional qualifications.

*“Very often you would like to do more, but you simply don‘t have the time. This really is a shame in this profession: we go through our day like a hamster on a wheel and we can only touch the surface. But when you do this very thoroughly you also are more satisfied.” (MPA_16).*

MPAs seem to be persons of trust to whom patients can talk to on their own level. In contrast, GPs are seen as authority figures. Accordingly, patients entrust MPAs with more intimate information. MPAs hereby undertake the important task of distinguishing between personal and health-related information and decide what to communicate to the GP.

*“For example, when you are alone with a patient while taking a blood sample, they take the opportunity to tell me things they wouldn’t want to report to the doctor.” (MPA_6).*

*“The doctor is seen almost as a god by some patients. So, we are rather on the same level and they rather share their worries with me.” (MPA_16).*

In line with the perspectives of the GPs, the MPAs were concerned that an additional coordinator or navigator from outside the practice might disturb the workflow.

*“Well, I think if we were better organised, we would be able to manage everything from within. Somebody from the outside would really need time to understand the processes, and probably every practice is a bit different.” (MPA_16).*

## Discussion

This paper explores the perspective of GPs, complemented by the views of MPAs of the same practices, of requirements and barriers for the coordination of care of patients with complex needs, such as multimorbid patients.

While the responsibility of the GPs for the coordination of patient care is embodied in German social law, in the interviews we found very heterogeneous views on what coordination entails, especially in the context of complex care for multimorbid patients.

A study from Krug H. demonstrates the necessity of additional expenditure for the care of elderly, multimorbid patients. New financial concepts should be adopted to prevent negligence occurring in the patients’ medical care [23].

Our results confirm the problem of the lack of adequate compensation for coordination tasks, which may hinder the GPs from giving optimal care to their patients. GPs have to consider their resources while trying to provide optimal care to their patients [24, 25].

A barrier discussed by many of the interviewed GPs and MPAs is the insufficient cooperation and communication between different players in the healthcare system, especially across the sectoral borders. Successful cooperation requires a full and timely flow of information.

The interviews show that the importance of cooperation across the sectors is particularly reflected in the problems regarding the discharge of the patient from hospital. Treatment reports and discharge letters were reported as often not reaching the GPs in time. Medication regimes as recommended by the hospital are often not fully understood by the patients at the time of discharge.

This is in line with previous research. An international health policy survey with primary care physicians from 2006 shows that more than 50% of primary care physicians wait over 14 days before receiving the patients’ full report from the hospital after their discharge. 70% of the responding GPs stated the importance of better integrating information systems between office-based physicians and hospitals [6]. Other studies confirm that discontinuation of care happens at the interface between the inpatient and outpatient sectors [23, 26–29]. Also Gulliford et al. confirm that problems with management continuity of care are highly associated with hospital utilisation [3]. The focus still is on acute medical care and specialist care. Too little emphasis has been given to life-long support for chronically ill patients in primary care [2]; life-long support measures are implemented in the general practice. A coordinated care in general practice including a good relationship between doctors and patients and the collaboration between different professions in different settings goes hand in hand with continuity of care [3].

Furthermore, statutory regulations require substantial time and effort for administrative and organisational matters. The documentation of work, filling in forms and administrative tasks are part of the doctors’ responsibilities.

The interviews with GPs confirm that these tasks are a major part of the coordination of care in general practice. The interviewed GPs burdened by assuming organisational and administrative tasks while providing medical care. Assigning responsibility for these tasks to someone else, such as an additional MPA, could allow them to focus on the medical care of the patients. The findings from our interviews are in line with the findings of a study from Margolius et al.; at times of primary care physician shortage, it may help to improve patients’ care by reorganising practices to expand the capacity for staff members to engage in different tasks [30].

While expressing a need for support and identifying possible tasks that could be delegated, GPs mostly seem to favour support by an additional healthcare professional who works within the general practice while retaining ultimate responsibility.

The findings from our interviews with MPAs confirm that they are highly motivated to undergo further training to take over more responsibilities and to relieve GPs. Previous studies also show that MPAs are interested in gaining supplementary qualifications. Further training of MPAs can strengthen the role of the MPA in the practice team and improve the quality of patients’ care as well as the satisfaction of patients [31, 32]. However, the expansion of roles in general practice will only be feasible if GPs do not feel threatened by the shift of territory and responsibility [15, 17].

As for coordination tasks, which were the focus of our study, the question arises as to whether one single, uniform concept for the support of the GPs would fit the setting of each individual general practice. The expert report from the ‘Advisory Council on the Assessment of Developments in the Healthcare System’ [2] underlined the need for further structural development in primary healthcare in Germany, involving a restructured distribution of work and competencies, in which non-medical professionals will take over more tasks and responsibility. The advisory council stressed the need for research on the significance of organisational features such as leadership and practice culture in Germany, as well as internal resources for changes on health outcomes in general practice [2].

GPs are highly aware of multimorbid patients’ unmet social needs and they do not feel sufficiently equipped and qualified to respond to questions concerning the social and legal sector. Another professional group – such as social workers or MPAs with further training in social and legal fields, in cooperation with other local institutions – could address social and legal issues without interfering with GPs’ responsibility. These findings are in line with previous studies. Social workers can provide comprehensive care, which goes hand in hand with a broad range of roles, such as, helping patients to cope with their complex chronic illness providing counselling, or supporting patients and families in their daily routines [33]. McGregor et al. found that patients with complex needs may obtain measurable health benefits from the deployment of social workers in primary care settings [34]. According to Zimmermann et al., cooperative and “low-threshold” solutions to employ non-medical support measures in the general practice are required [35].

### Strengths and limitations

Certain limitations should be taken into account when interpreting the findings of the study.

Recruiting GPs from a practice-based research network assures sufficient participation rates but might introduce sampling effects and bias by selecting GPs who are interested in research and therefore more committed than others [36]. However, the non-random selection of participants by applying a purposive sampling strategy is the adequate choice when aiming at understanding the opinion of individuals who are already informed or interested in the topic of the study [37].

We believe that by performing a comprehensive survey as the next step in the project, on the basis of the results of the qualitative interviews, a broader perspective of a large number of GPs will be integrated with the deeper insights of a selected group. By using the framework approach to analyse the data, we could benefit from multidisciplinary teamwork. This approach may also add different perspectives and provide more depth to the data.

The patient perspective will be evaluated in further subprojects: an ongoing interview study explores the view of chronically ill patients on the quality of care and unmet needs. Furthermore, as part of a regular nationwide survey members of the public are asked for their view on delegation of tasks to medical practice assistants. The results will complement the perspective of GPs and MPAs presented here.

There may also be limitations concerning the transferability of the results to other countries. The German healthcare system neither has a gatekeeping system nor rules for committing patients to specific practices. Our findings reflect the characteristics of German settings. Nonetheless, we discussed and underpinned our findings with several studies from the international literature, in which new models of coordination in general practice have already been developed.

## Conclusions

Coordination is a primary component in patient care in general practice. The term “coordination” includes a wide range of tasks, which vary depending on the structure of the practice. The GP, as the coordinator of the whole process of care, has the main responsibility. Therefore, they also take responsibility for tasks which aren’t exclusively medical and are not remunerated. The support of patients with multimorbidity, in particular, is comprehensive and involves players across different sectors. In order to ensure optimal care for this patient group, the general practice needs to obtain coordinative support. The findings from the interviews in this study show that GPs and MPAs hold diverse opinions about possible solutions. Both GPs and MPAs are generally interested in implementing new concepts, but they are also still sceptical about the practicability. GPs are nevertheless more open to the deployment of an additional healthcare professional within the practice rather than from outside.

## Supplementary information

**Additional file 1: Figure S1.** NAVICARE network. Authors own figure of NAVICARE project structure

## Data Availability

The data in this paper is based on the transcripts of 16 audio-recorded interviews with GPs and their MPAs. Data supporting the findings of this study can be found in the translated quotes in the results section of this article. However, to protect the participants’ identities, the full data from this study (transcripts and audio files) will not be made available to the public.

## Abbreviations

DMP: Disease management programs
GP: General Practitioner
MPA: Medical practice assistant
