# Supplementary material for: Coordination of care for multimorbid patients from the perspective of general practitioners – a qualitative study
Source: BMC Fam Pract. 2019 Nov 20;20:160. doi: 10.1186/s12875-019-1048-y (PMC6865037; doi:10.1186/s12875-019-1048-y)
Supplement: Supplementary file 1 — Additional file 1: Figure S1. NAVICARE network. Authors own figure of NAVICARE project structure [file 12875_2019_1048_MOESM1_ESM.docx]

Supplementary Figure 1: NAVICARE network


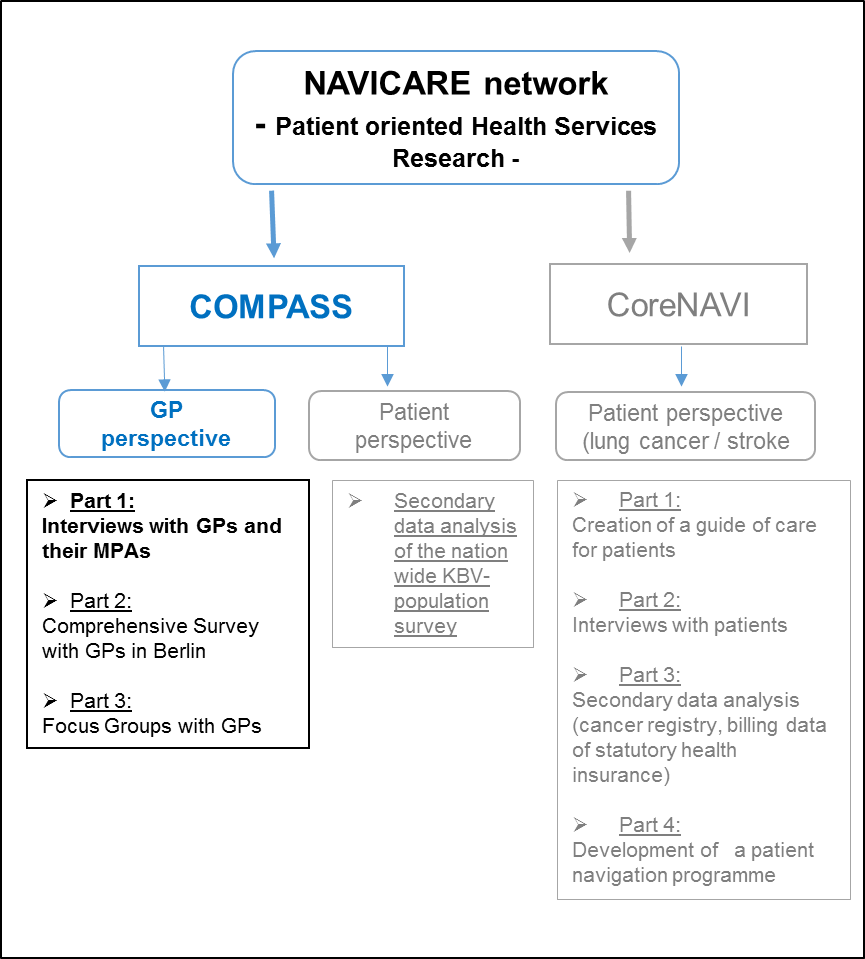


*Supplementary Figure 1: Authors’ own figure of NAVICARE project structure*
